# Bone flap resorption and its risk factors following autologous cranioplasty with bone tissue bank–stored grafts

**DOI:** 10.1007/s10143-026-04515-3

**Published:** 2026-10-03

**Authors:** Xiaoliang Du, Yonggang Liu, Weijun Qiao, Lexiao Feng, Xingxing Wu, Zhen Chen, Yongyong Su, Zhenfeng Liu, Zeyuan Sun, Xiaojuan Wang, Linlin Zhang

**Affiliations:** 1 Department of Neurosurgery, Xianyang Central Hospital, Xianyang, Shaanxi 712000 China; 2 Ward I & Palliative Care Unit, Cancer Center, Xianyang Central Hospital, Xianyang, Shaanxi 712000 China; 3 Department of Medical Imaging, Xianyang Central Hospital, Xianyang, Shaanxi 712000 China; 4 Department of Critical Care Medicine II, Xianyang Central Hospital, Xianyang, Shaanxi 712000 China

**Keywords:** Bone tissue bank, Autograft, Cranioplasty, Bone flap resorption, Risk factors

## Abstract

To classify the severity of bone flap resorption (BFR) following autologous cranioplasty under bone tissue bank storage conditions and to identify potential risk factors by analyzing clinical cases exhibiting postoperative bone resorption. A retrospective analysis was conducted on clinical data from 61 patients who developed bone flap resorption after autologous cranioplasty performed with bone flaps stored under bone tissue bank conditions. Based on the Oulu Resorption Scale, patients were categorized into four groups: no bone flap resorption (NBFR), mild BFR (MBFR), intermediate BFR (IBFR), and severe BFR (SBFR). Clinical characteristics were compared across these groups. Variables showing statistical significance (*p* < 0.05) in univariate analyses were subsequently entered into a multivariate ordinal logistic regression model to evaluate their association with the development and severity of BFR. All 61 patients completed follow-up, with a median follow-up duration of 378.0 days (interquartile range: 178.5–605.5 days). By the end of follow-up, 16 patients (26.2%) had developed BFR, including 9 cases (14.8%) of MBFR, 4 cases (6.6%) of IBFR, and 3 cases (4.9%) of SBFR. Univariate analysis revealed that smoking Pre- decompressive craniectomy (Pre-DC), traumatic brain injury (TBI), number of bone flaps ≥ 2, cranial defect area > 92.11 cm², and bone flaps located in the frontotemporoparietal region were significantly associated with an increased risk of BFR (all *p* < 0.05). Multivariate ordinal logistic regression analysis identified smoking Pre-DC, TBI, and cranial defect area > 92.11 cm² as independent risk factors for BFR, whereas having fewer than two bone flaps served as a protective factor against resorption (all *p* < 0.05). Autologous cranioplasty using bone flaps preserved under bone tissue bank conditions is associated with a low complication rate. With careful patient selection, it remains an admissible reconstructive option following decompressive craniectomy.

## Introduction

Decompressive craniectomy is a common neurosurgical procedure primarily indicated for conditions such as cerebral trauma, extensive cerebral infarction, intracerebral hemorrhage, and brain tumors. Postoperatively, most patients undergo cranioplasty depending on their recovery progress. Currently, materials available for cranial reconstruction include autologous bone grafts, polyetheretherketone (PEEK), titanium mesh, bone cement, hydroxyapatite, 3D porous titanium, and high-molecular-weight polyethylene, each with distinct advantages and disadvantages. When considering cosmetic outcomes, rejection rates, economic cost, and patient acceptance, autologous bone is often favored [1–3], however, bone flap resorption (BFR) remains a major concern.

The diagnostic criteria for BFR have not been fully standardized [4], reported incidence rates range widely from 3.6% to 90%, and its risk factors remain controversial [3, 5–14]. Therefore, based on the Oulu Resorption Scoring Scale [9] proposed by Korhonen et al. (Table 1), we classified BFR into different grades and investigated the associated risk factors, aiming to provide evidence for the broader application of autologous bone grafts.

**Table 1:** The Oulu resorption score proposed by Korhonen, et al. [9]

Variable	Description	Score
Extent	Remaining bone volume	
	No BFR/remaining bone volume = 100.0%	0
	Remaining bone volume 75.0 to 99.9%	0
	Remaining bone volume 25.0 to 74.9%	2
	Remaining bone volume < 25.0%	3
Severity	Perforations due to BFR	
	No BFR or only cancellous bone loss	0
	Non-perforating resorption	1
	A new bi-cortical perforation of < 1.0 cm	2
	A new bi-cortical perforation of ≥ 1.0 cm	3
Focus	Total integrity of the bone flap	
	No BFR	0
	One focal BFR change	0
	Multiple BFR foci	2
	Diffuse BFR: signs of BFR throughout the flap area	3

Σ the sum of the Extent, Severity, and Focus scores, making up the Oulu resorption score.

## Methods

### Patient population

We performed a retrospective study of patients who underwent cranioplasty used autologous bone flap from September 1,2022 to September 30,2025, experienced decompressive craniectomy, previously. Of course, patients who required subsequent removal of the bone flap due to infection following cranioplasty were excluded from this study.

### Clinical and Radiological data

We collected their clinical data according medical records, such as gender, age, history of smoking Pre- decompressive craniectomy (Pre-DC), etiology of craniectomy (traumatic brain injury/intracerebral hemorrhage/cerebral infarction/ruptured cerebral aneurysm), the area of bone defect, presence of fracture, number of bone flaps, location of bone flaps, time interval between decompressive craniectomy and cranioplasty (duration of bone flap preservation), preoperative Glasgow Outcome Scale (GOS) score, postoperative follow-up duration and imaging data of Computed tomography from Picture Archiving and Communication Systems(PACS), and then measured the size of the skull defect using the 3D Slicer software (version 4.11).

### Bone flap sterilization, preservation, storage and reimplantation

After decompressive craniectomy, surface contaminants on the bone flap were thoroughly removed, and the flap was sealed in a sterile bag. It was then immediately placed in a −80 °C environment and transferred to the bone tissue bank for sterilization using gamma irradiation. A Cobalt-60 (⁶⁰Co) source was employed as the radiation source. The samples were subjected to a total absorbed dose of 25 kGy (kilogray) at room temperature. This “cold” sterilization method was chosen to ensure the complete inactivation of bacteria, viruses, and fungi by damaging their nucleic acids, while preserving the structural and functional integrity of the material. The sterilized bone flaps were stored in a bone tissue bank at −80 °C. When bone flap reimplantation was required, the flap was retrieved from the bone tissue bank, thawed at room temperature in normal saline for 30 min, and then cranial repair surgery was performed.

### Modified Oulu Grouping

All patients with BFR were scored according to the Oulu resorption score and reclassified, given the heterogeneity within the category that had a total score of 0 in this classification system. Specifically, an Oulu score of 0 can represent either perfect bone preservation or minor changes. To improve sensitivity, we defined the MBFR group to include these early radiological signs, distinguishing them from the NBFR group [strictly defined as a extent score of 0 (No BFR/remaining bone volume = 100.0%) + a Severity score of 0 (No BFR) + a Focus score of 0 (No BFR)]. In other words, this study reclassified patients according to the Oulu Resorption Score into the following groups (Table 2): No Bone Flap Resorption (NBFR) group [Oulu Resorption Score of 0, specifically defined as a extent score of 0 (No BFR/remaining bone volume = 100.0%) + a Severity score of 0 (No BFR) + a Focus score of 0 (No BFR)], Mild Bone Flap Resorption (MBFR) group (Oulu Resorption Score of 0–4, excluding patients meeting the NBFR criteria with a total score of 0), Intermediate Bone Flap Resorption (IBFR) group (Oulu Resorption Score of 5–8), and Severe Bone Flap Resorption (SBFR) group (Oulu Resorption Score of 9).

**Table 2:** Comparison of the Oulu grade with the modified Oulu group in our study

Score	Oulu grade	modified Oulu group
0	0	NBFR/MBFR MBFR
0< Score ≤ 4	Ⅰ	
4< Score ≤ 8	Ⅱ	IBFR
9	Ⅲ	SBFR

### Statistical analysis

Normally distributed numerical data were expressed as mean ± standard deviation (x̄ ± s), and intergroup comparisons were performed using one-way analysis of variance (ANOVA). Non-normally distributed numerical data were expressed as median and interquartile range, and intergroup comparisons were performed using the Kruskal-Wallis non-parametric test. Categorical data were expressed as frequency (percentage), and intergroup comparisons were performed using the chi-square test. A *p* < 0.05 was considered statistically significant. For variables with *p* < 0.05, post-hoc tests were conducted using the Bonferroni method. Numerical and categorical variables with *p* < 0.05 in the univariate analysis were included in a multivariate ordinal logistic regression analysis, where *p* < 0.05 was considered statistically significant. Analyses were performed using the IBM Statistical Package for the Social Sciences, version 26.0 (SPSS Inc., Chicago, IL, USA).

## Results

### Baseline characteristics

Sixty-one patients (One patient developed an infection after the surgery, so excluded from this study) included in this study had undergone primary autologous cranioplasty after decompressive craniectomy. The baseline characteristics are reported in Table 3, there were 46(75.4%) men and 15(25.6%) women, mean patient ages was 52.33 ± 11.49 years. Fifty-seven patients (93.4%) scored GOS 4–5 and four (6.6%) scored GOS 2–3. Nineteen patients (31.1%) had a history of smoking Pre- DC. Thirteen patients (21.3%) had decompressive craniectomy for intracerebral hemorrhages, 27 (44.3%) had trauma, 12 (19.7%) had cerebral infarctions, 9 (14.7%) had Aneurysmal subarachnoid hemorrhage. The median interval from craniectomy to cranioplasty was 124.0days, the median interval from cranioplasty to evaluated BFR was 378.0 days. Forty-three patients (70.5%) had less 2 bone flaps and 18 (29.5%) more then 2. The median area of bone defect was 80.93cm [2]. Regarding the location of skull bone flap removal, there were 11 cases (18.0%) in the frontotemporal region, 40 cases (65.6%) in the frontal-temporoparietal region, 9 cases (14.8%) in the temporoparietal region, and 1 case (1.6%) in the occipitoparietal region. During the follow-up period, no patient underwent ventriculoperitoneal (VP) shunt placement. Five patients developed hydrocephalus but were managed conservatively; these cases were distributed as follows: 3 in the NBFR, 1 in the MBFR, and 1 in the SBFR. Due to the limited number of events, statistical comparison was not performed.

**Table 3:** Univariate statistical analysis of patients who underwent cranioplasty with autologous bone. ^*^ derived from the chi-square test; ^▲^derived from one-way analysis of variance (ANOVA); ^●^derived from the Kruskal-Wallis non-parametric test for multiple group comparisons. Subscript letters indicate whether differences exist for the same variable between different groups. When the letters are the same, it indicates no statistically significant difference; when the letters are different, it indicates a statistically significant difference. ICH Intracranial hemorrhage; TBI Traumatic brain injury; AIS Acute ischemic stroke; SAH Subarachnoid hemorrhage; n number; DC decompressive craniectomy

Variable	Total	NBFR		BFR		*P* Value
			MBFR	IBFR	SBFR	
Gender, n (%)						0.511༊
Male	46(75.4%)	32(71.7%)	8(88.9%)	3(75%)	3(100%)	
Female	15(24.6%)	13(28.3%)	1(11.1%)	1(25%)	0	
Age (years)	52.33 ± 11.49	51.89 ± 12.37	56.44 ± 7.23	51.25 ± 11.84	48.00 ± 7.81	0.651^▲^
Pre-cranioplasty GOS score, n (%)						0.677༊
4–5	57(93.4%)	41(91.1%)	9(100%)	4(100%)	3(100%)	
2–3	4(6.6%)	4(8.9%)	0	0	0	
Smoking (Pre-DC), n (%)	19(31.1%)	8(17.8%)b	5(55.6%)a	3(75%)a	3(100%)b	0.001༊
Reason for DC, n (%)						0.029༊
ICH	13(21.3%)	12(26.7%)a	1(11.1%)a	0 a	0 a	
TBI	27(44.3%)	13(28.9%)b	8(88.9%)a	3(75%)a	3(100%)a	
AIS	12(19.7%)	12(26.7%)a	0 a	0 a	0 a	
SAH	9(14.7%)	8(17.7%)a	0 a	1(25%)a	0 a	
Interval time between DC and cranioplasty, n (%)						0.642༊
≤ 90	5(8.2%)	3(6.7%)	2(22.2%)	0	0	
91–180	40(65.6%)	31(68.9%)	5(55.6%)	2(50%)	2(66.7%)	
>180	16(26.2%)	11(24.4%)	2(22.2%)	2(50%)	1(33.3%)	
Bone flap, n (%)						<0.001༊
<2	43(70.5%)	38 (84.4%) a	4(44.4%)a	1(25%)a	0 a	
≥ 2	18(29.5%)	7(15.6%)b	5(55.6%)a	3(75%)b	3(100%)b	
Bone defect(cm^2^)	80.93(56.28,96.47)	68.23(50.81,90.46) a	92.11(88.92,105.08) b	98.22(68.55,132.10) a	109.6(56.44,109.6) a	0.014^●^
Location of bone flap, n (%)						0.001༊
frontotemporal	11(18.0%)	7(15.5%)a	4(44.4%)a	0 a	0 a	
frontal-temporal parietal	40(65.6%)	30(66.7%)a	4(44.4%)a	4(100%)b	2(66.7%)a	
temporoparietal	9(14.8%)	8(17.8%)a	1(11.2%)a	0 a	0 a	
occipitotemporal	1(1.6%)	0 a	0 a	0 a	1(33.3%)a	

### Statistical results

**Fig. 1:**
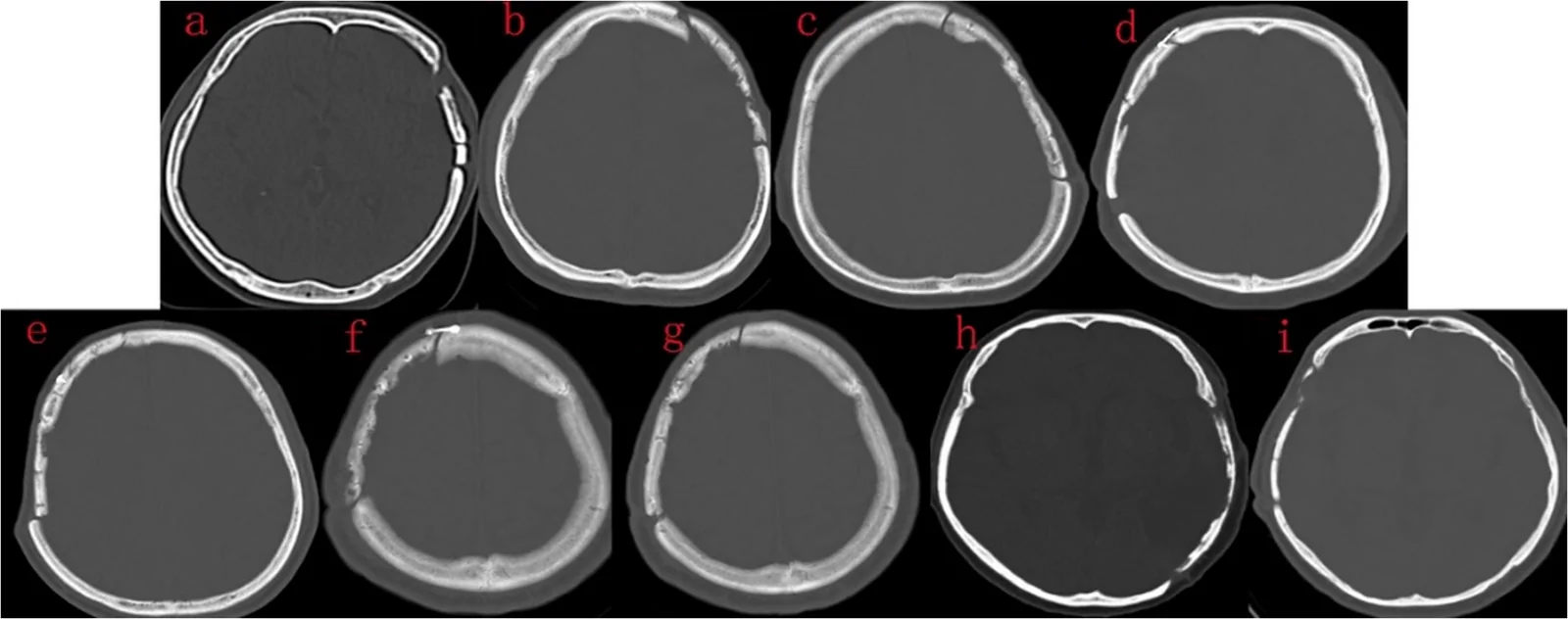
9 cases of MBFR, which Oulu Resorption Score of 0–4(2,2,1,2,1,4,3,4, and 2, respectively). In these nine patients, BFR occurred at 226, 375, 491, 320, 158, 834, 16, 471, and 156 days post- cranioplasty, respectively

**Fig. 2:**
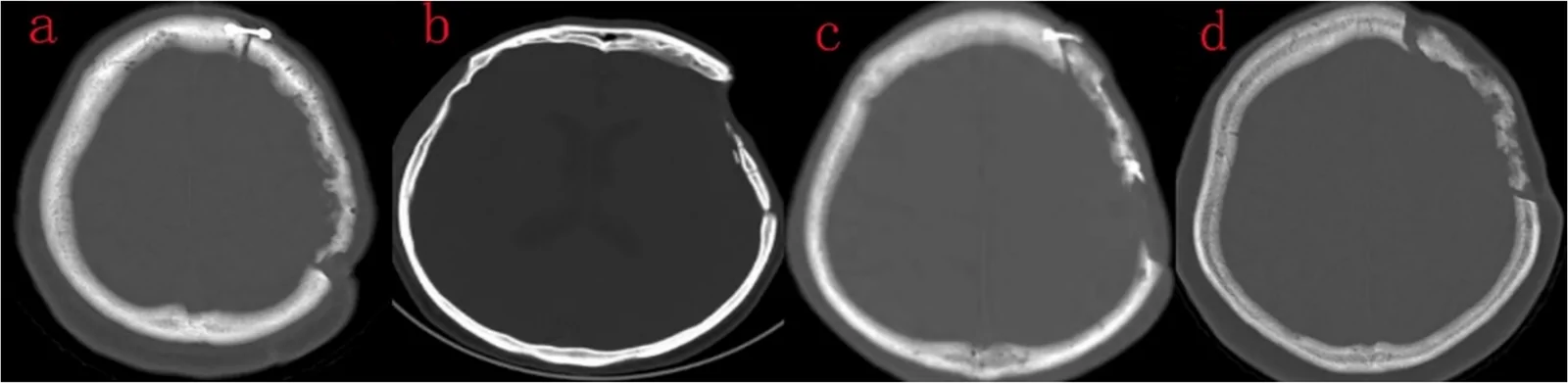
4 cases of IBFR, which Oulu Resorption Score of 5–8(5,7,7, and 5, respectively). In these four patients, BFR occurred at 438,1004,161, and 514 days post- cranioplasty, respectively

**Fig. 3:**
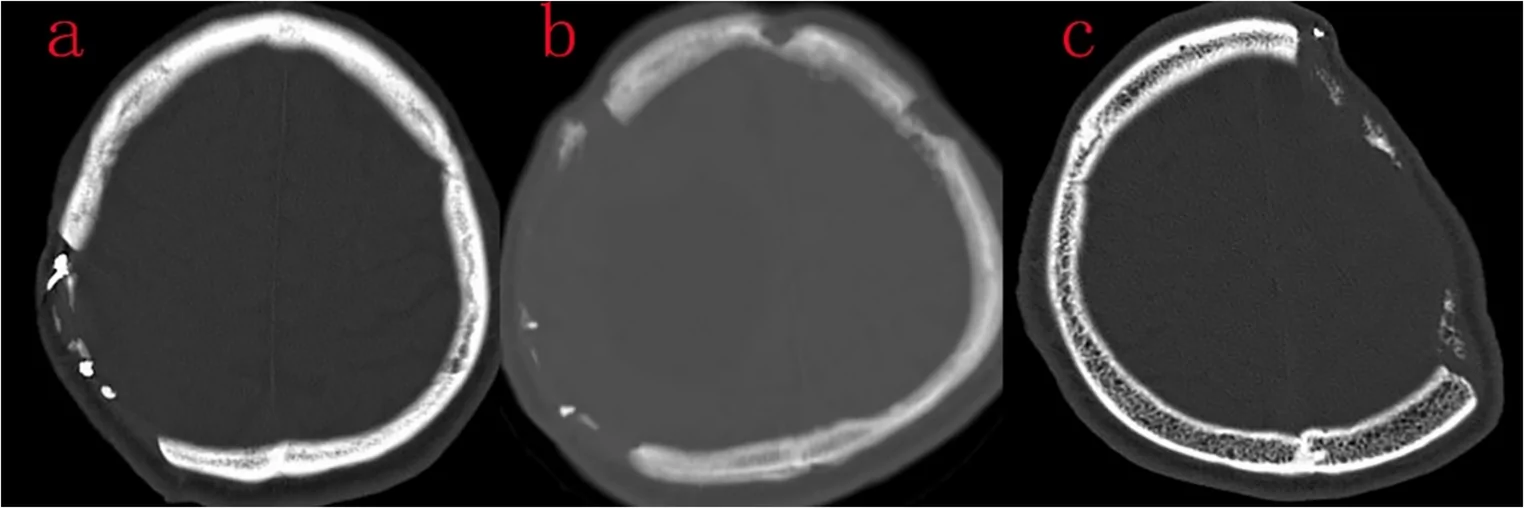
3 cases of SBFR, which Oulu Resorption Score of 9. In these three patients, BFR occurred at 387,167, and 355 days post- cranioplasty, respectively

**Fig. 4:**
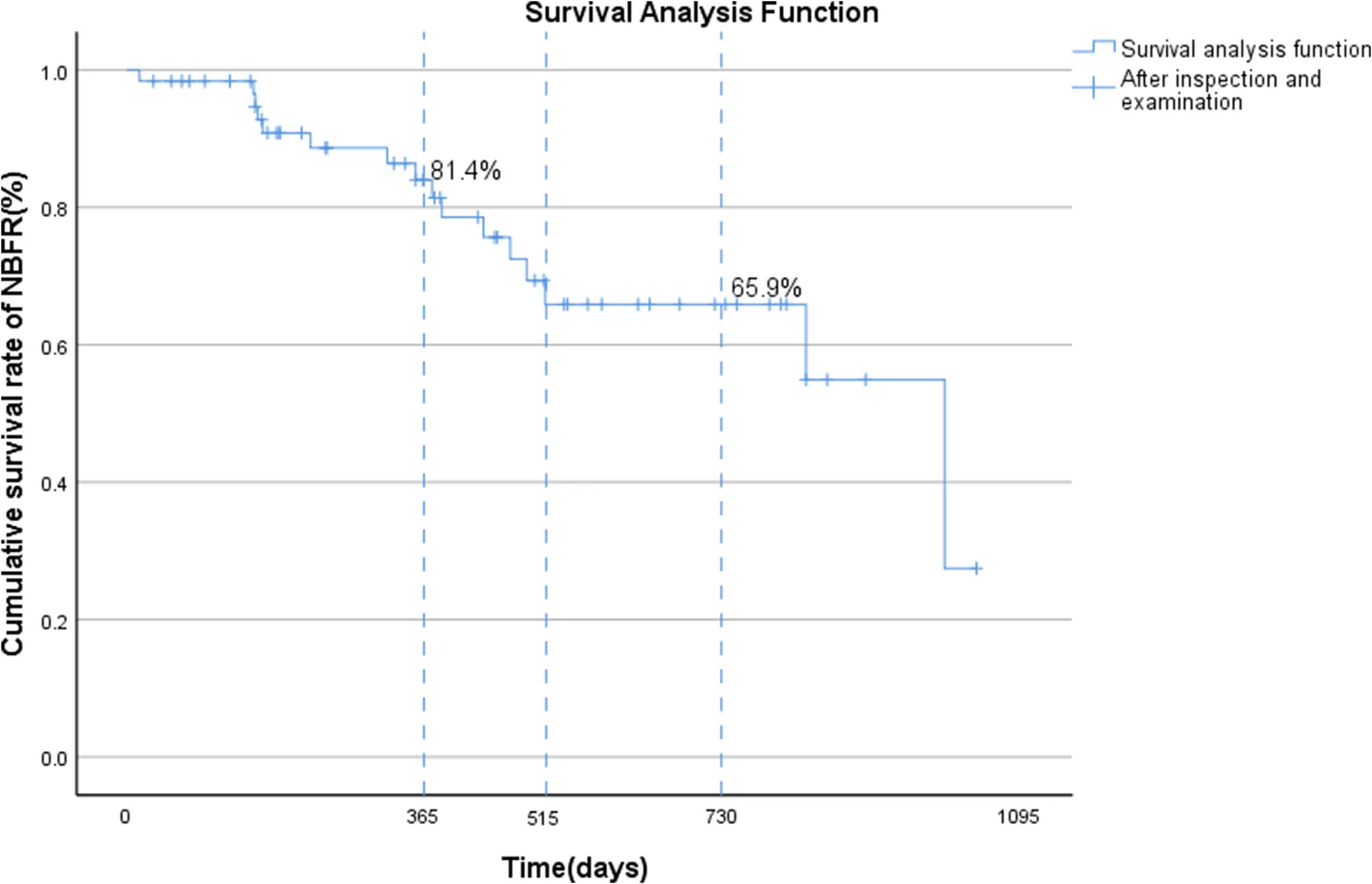
Kaplan-Meier survival curve for BFR after autologous cranioplasty. The curve shows the cumulative rate of freedom from any degree of BFR in the entire cohort (*n* = 61). Ticks indicate censored cases. Vertical dashed lines represent 1 year (365 days) and 2 years (730 days) after surgery, with corresponding survival rates of 81.4% and 65.9%, respectively. Note the plateau in the curve after 515 days, indicating a reduced hazard rate in the long term

Among all 61 patients, 45 cases (73.8%) were classified as NBFR, and 16 cases (26.2%) as BFR, which included 9 cases (14.8%) of MBFR, 4 cases (6.5%) of IBFR, and 3 cases (4.9%) of SBFR (Table 3). Among the 16 patients with BFR, it occurred at an average of 379 days after cranioplasty, with the earliest observed at 16 days and the latest at 1004 days (Fig. 1/Fig. 2/Fig. 3). The latest occurrence of SBFR was observed at 387 days after cranioplasty, with a mean time of 303 days (Fig. 3). The cumulative incidence of BFR at 1 and 2 years after cranioplasty was 18.6% and 34.1%, respectively. The survival curve plateaued significantly after 515 days, suggesting that the risk of developing new, clinically significant resorption diminishes after the first 1.5 years. (Fig. 4). Univariate analysis (Table 3) showed that history of smoking Pre-DC, traumatic brain injury (TBI), the presence of ≥ 2 bone flaps, larger bone window area, and frontal-temporoparietal bone flap location were significantly associated with a higher incidence of BFR (*p* = 0.001, *p* = 0.029, *p* = < 0.001, *p* = 0.014, and *p* = 0.001, respectively). Post-hoc analysis indicated that, compared to the NBFR group, smokers were more prone to developing MBFR and IBFR; TBI patients were more susceptible to MBFR, IBFR, and SBFR; having ≥ 2 bone flaps was more likely to lead to MBFR; a larger bone window area was associated with a higher risk of MBFR; and frontal-temporoparietal bone flap location was more frequently linked to IBFR. Multivariate ordinal logistic regression analysis (Table 4) identified smoking, TBI, and a bone window area > 92.11 cm² as independent risk factors for BFR, while having < 2 bone flaps served as a protective factor against BFR (*p* = < 0.001, *p* = 0.045, *p* = 0.002, and *p* = < 0.001, respectively).

**Table 4:** Multivariate ordered logistic regression analysis of bone resorption

Variable	β	SE	Wald x^2^	95% CI	OR	*P* value
Smoking (Pre-DC)	3.823	1.037	13.583	1.790 ~ 5.855	45.74	<0.001
TBI	2.922	1.512	3.985	0.162 ~ 6.025	18.58	0.045
Location of bone flap	−1.268	1.344	0.890	−3.903 ~ 0.366	0.28	0.345
Bone defect>92.11cm^2^	0.038	0.012	9.915	0.014 ~ 0.062	1.04	0.002
Bone flap<2	−2.829	0.698	16.419	−4.198 ~ 1.461	0.06	<0.001

## Discussion

Autologous cranioplasty is a common method for skull reconstruction following decompressive craniectomy, with a long history of clinical application and mature techniques. However, the high incidence of complications, such as bone resorption and bone flap infection, following reconstruction has driven continuous exploration of cranial bone preservation methods in clinical practice. These include high-temperature approaches such as alcohol immersion, autoclaving, and calcination [15]. Such methods, however, significantly denature bone morphogenetic proteins and alter the collagen matrix, potentially reducing the osteoinductive capacity of the bone flap and increasing its resorption rate [16]. As a result, they are rarely considered among available clinical options. Another strategy involves preserving bone cell viability, such as subcutaneous preservation and cryopreservation, the former requires additional abdominal surgery for both preservation and retrieval of the bone flap, which may cause morphological changes to the abdomen, increase abdominal discomfort, and prolong hospital stay [17]—factors that have led to its gradual discontinuation in clinical practice. The latter, that is, the cryopreservation including preserved at −18 °C, although more economical and without added patient discomfort, this approach is associated with higher rates of bone flap resorption and postoperative infection [17, 18]. And at liquid nitrogen preservation (−196 °C), involves a complex processing protocol for the bone flap. Any errors during preservation or pre-operative thawing can induce severe cytotoxicity and pose a high risk of bone resorption. These limitations have restricted the clinical application of this preservation method [15]. In contrast, the bone flaps examined in this study were all preserved according to the standard bone tissue bank protocol, which involves storage at −80 °C and sterilization with cobalt-60 irradiation. This protocol aims to minimize infection risk while better preserving the biological activity of bone cells and structural proteins [19]. In our study, only one patient developed a postoperative infection, the infection rate was 1.6%, which is substantially lower than that reported for other preservation methods [7, 17, 18]. It indicates the complication of infection after Autologous cranioplasty which under the preservation conditions of the bone tissue bank is relatively reduced.

Previous studies have reported that the incidence of bone flap resorption (BFR) following autologous cranioplasty ranges from 3.6% to 90%^3, 5–8, 20^. This wide variation may be attributed to the lack of a unified diagnostic standard for BFR [3–9]. In recent years, Korhonen et al. ^[9]^ proposed a grading and classification system for BFR based on cranial computed tomography (CT), categorizing it into non-severe and severe bone resorption. However, in this grading system, patients classified as Oulu grade 0 were not strictly distinguished but were uniformly included in the non-severe resorption group. Consequently, the reported incidence of BFR was significantly inflated. Excluding that particular study, most research indicates a BFR rate of 3.6% to 26.9%. Building upon the Oulu resorption scoring scale proposed by Korhonen et al., this study is the first to propose a reclassification of patients with an Oulu grade of 0. The aim is to more accurately reflect the true incidence of BFR and to further analyze its potential associated factors. Following reclassification, our results indicated that the cumulative incidence of BFR during the follow-up period was 26.2%, with the rate of SBFR being 4.9%. Further survival analysis revealed that the probability of BFR occurrence at 1 year and 2 years after autologous cranioplasty was 18.6% and 34.1%, respectively. The risk of BFR significantly decreased after 1.5 years. Notably, SBFR occurred on average within 303 days postoperatively. Therefore, although BFR may still occur after the first year, it predominantly manifests as MBFR or IBFR. In summary, we conclude that the first year of follow-up after autologous cranioplasty is critical. The “critical window period” for severe, clinically significant BFR is concentrated within the first year. Nevertheless, longer follow-up remains valuable as it facilitates the detection of a greater number of MBFR and IBFR cases.

One study found that after the bone flap was repositioned, the medullary cavity was filled with fibrous connective tissue and necrotic components. The old osteoblasts exhibited no cellular activity, while osteoclasts became active, initiating bone resorption. After a period, new osteoblasts within the bone flap, guided by the old osteoblasts, initiated bone remodeling [20]. Subsequently, bone resorption gradually stabilized. During this process, smoking may affect postoperative outcomes through multiple pathways [3, 10, 20, 21]: The specific mechanisms likely involve cadmium and nicotine, among other tobacco constituents, which promote osteoclastogenesis and suppress osteoblast activity, thereby decreasing cortical bone density and thickness and contributing to bone resorption. In this study, univariate and multivariate analyses revealed a significant association between smoking and the occurrence of bone resorption. Therefore, patients with a long-term smoking history should be informed of their significantly increased risk of bone resorption. For patients who have difficulty quitting smoking, the use of artificial cranial repair materials is recommended when necessary.

In patients undergoing cranioplasty, the incidence of bone resorption is higher when the primary disease is traumatic brain injury (TBI) compared to other etiologies [3, 11, 12]. This association may be attributed to the following reasons: Firstly, inflammatory response: trauma patients often exhibit a systemic inflammatory response, and inflammatory cytokines promote osteoclast differentiation; Then, fracture occurrence: trauma patients may sustain fractures, which can relatively impede the osteogenic process and increase the risk of bone resorption; Finally, patients with TBI are generally younger than those with other etiologies. However, some researchers have not observed a significant difference between the two groups [7]. In this study, univariate and multivariate analyses identified TBI as an independent risk factor for bone resorption. Nevertheless, as TBI accounted for a high proportion of the study population, this factor requires further validation with larger datasets.

This study found that a bone flap count of less than two was a protective factor against bone resorption. Previous studies have demonstrated a significant increase in bone resorption with an increasing number of bone flaps. This correlation is believed to be associated with the following reasons [3, 14, 22, 23]: Firstly, an increased number of bone flaps enhances the barrier effect of fatty bone flaps (i.e., grafted bone flaps filled with fibrous connective tissue and necrotic components within the medullary cavity), leading to slower generation of new osteoblasts and consequently enhanced bone resorption. Secondly, an increased number of bone flaps results in a greater quantity of osteoclasts derived from cancellous bone entering the systemic circulation, thereby promoting bone resorption. Thirdly, an increased number of bone flaps compromises fixation stability, impairs bone fusion, and elevates the risk of resorption. Finally, an increased number of bone flaps indicates that the skull has sustained substantial external force, resulting in severe local damage and interference with bone fusion. Therefore, when surgically feasible, preserving the bone flap as a single piece is associated with better outcomes, which is consistent with the principle of minimizing surgical trauma.

The primary objective of decompressive craniectomy is to remove a sufficient area of the skull to achieve adequate decompression. However, this study found that when the bone window area is ≥ 92.11 cm², the risk of bone resorption following autologous bone flap repair is significantly higher. Similar findings have been reported in previous studies [3, 12, 13], although the critical bone window area varies across different studies. The underlying reasons are analyzed as follows: First of all, a larger bone window area results in a limited “bridging” effect from the old osteoblasts located at the bone margins, leading to slower bone reconstruction and a higher susceptibility to bone flap resorption (BFR). Secondly, a larger bone window area releases a greater number of osteoclasts, relatively increasing the likelihood of BFR. Lastly, a larger bone window area requires more time for the formation of blood vessels that nourish the bone flap, resulting in slower vascular reconstruction and a higher propensity for BFR. Therefore, considering both the surgical objective and the findings of this study, the following recommendations are proposed for patients scheduled for skull bone flap removal: preoperative planning of the surgical incision should be thorough and rational; intraoperatively, the periosteum-attached bone flap should be preserved as much as possible, and the use of materials such as bone wax that may impede “bone bridge” formation should be minimized; blind and extensive cauterization of the dura mater should be avoided; if an artificial dura mater is required, one of appropriate size and with good seam integrity should be selected; postoperative wound management should be emphasized to reduce inflammation. If the area of skull removal is excessively large, the use of other artificial materials to repair the cranial defect is recommended when necessary.

### Strengths and Weaknesses

Given the retrospective nature of this study, certain limitations are inherent. Incomplete historical records precluded the uniform application of a standardized clinical scoring system (e.g., a flap integrity score) across all 61 patients over the three-year period. As isolated radiological findings are insufficient for clinical decision-making, we caution against their use as the sole criterion for surgical intervention without clinical correlation. In the univariate analysis, frontotemporoparietal bone flaps were associated with a higher likelihood of IBFR, but this association did not reach statistical significance in the multivariate regression analysis. This discrepancy may reflect differences in scalp blood supply and mechanical stress across anatomical sites, which requires validation in larger cohorts. Although previous studies have identified younger age as a risk factor for BFR, no significant correlation was observed in our study; this null finding, as well as the loss of significance for other variables in the multivariate model, should be interpreted cautiously and may be attributable to the limited sample size or specific demographic characteristics of our population. Moreover, the relatively small number of BFR events reduced the statistical power of the multivariate analysis and increased the risk of overfitting, as reflected by the wide confidence intervals for some odds ratios.

### Future Direction

Future large-scale, multicenter prospective studies that integrate radiological assessments with standardized clinical scoring systems are warranted to validate these findings and to develop a more robust composite scoring system to guide clinical decision-making. To reduce the occurrence of BFR, future approaches may include modifying the preservation environment of bone flaps, developing new biomaterials, applying tissue engineering techniques, utilizing stem cell delivery, and employing mechanical stimulation [14, 24].

## Conclusions

In summary, based on the Oulu absorption scoring scale (Korhonen et al.), BFR was reclassified into MBFR, IBFR, and SBFR. Analysis of limited clinical data showed that SBFR after autologous cranioplasty occurs predominantly within the first postoperative year, though long-term follow-up may detect more cases. The overall complication rate was low, supporting the feasibility of this technique. Smoking, traumatic brain injury, and a bone defect area > 92.11 cm² were independent risk factors for BFR, whereas having fewer than two bone flaps was inversely associated with BFR. However, the small sample size and single-center retrospective design may introduce bias.

## Data Availability

No datasets were generated or analysed during the current study.
